# Physical activity as a protective factor in the mood of children and adolescents: association with overweight and obesity

**DOI:** 10.3389/fped.2025.1494998

**Published:** 2025-03-20

**Authors:** Alexandro Andrade, Keyla Mara dos Santos, Anderson D’Oliveira, Verônica Maria Claudino, Whyllerton Mayron da Cruz

**Affiliations:** Sports and Exercise Psychology Laboratory—LAPE, Center for Health and Sports Sciences—CEFID, Santa Catarina State University—UDESC, Florianópolis, Brazil

**Keywords:** child nutrition disorders, adolescent, mental health, exercise, physical inactivity

## Abstract

**Introduction:**

Sedentary children and adolescents are more susceptible to developing diseases, obesity and psychological disorders, but little is known about the relationship between physical activity (PA), body mass index (BMI) and mood state in this population.

**Methods:**

This is a cross—sectional study with the participation of 2,757 students, aged between 10 and 19 years. The Brunel Mood Scale—BRUMS was used to assess the dimensions of tension, depression, anger, vigor, fatigue and mental confusion, and the PA questionnaire for children and adolescents was used to assess the practice of PA.

**Results:**

There was a significant difference between the eutrophic and overweight BMI groups for the mood states of depression (*p* = 0.004) and vigor (*p* = 0.047). On the other hand, the overweight (4.06 ± 4.3) and obese (3.85 ± 4.0) groups had the highest rates of depression. It was observed that a higher BMI was associated with depression, anger and fatigue in children and adolescents and that PA can explain 39% of vigor in this population (F = 103.062, *p* = 0.000; *R*^2^ = 0.039). Active children and adolescents had twice as much vigor as inactive ones. There were differences between males and females, and between active and inactive individuals, in depression, anger, vigor, fatigue and confusion. There was a tendency for overweight and obese children and adolescents to have higher rates of depression when compared to underweight or normal-weight individuals.

**Conclusion:**

The practice of physical activity (PA) was shown to have a positive effect on mood, with active children and adolescents reporting lower levels of depression, anger, fatigue, and confusion, and higher levels of vigor. These findings underscore the importance of PA as a protective factor against mood disturbances in this population, highlighting its potential role in improving emotional well-being.

## 1 Introduction

Physical activity (PA) is widely recognized for its health benefits in children and adolescents (1). However, 81% of adolescents aged 11–17 worldwide do not meet sufficient physical activity levels, with significant differences between sexes, regions, and countries (2). Physical inactivity represents a serious threat to the health and well-being of the general population, as these habits tend to persist into adulthood, with negative impacts on both public health and the economy (1–4).

In general, PA is recommended for children and adolescents, aiming to improve health in both the short and long term (5). Studies have shown a high incidence of PA absence and increased sedentary behaviors in children and adolescents, with significant implications for body composition (3, 4, 6, 7). Along with assessing PA levels, the body mass index (BMI) is a simple parameter, determined by the weight-to-height ratio, used to classify quantitative data in the general population, including for obesity classification (8).

Obesity and overweight are critical factors in the development of cardiovascular diseases, with alarming data showing approximately 17.8 million deaths annually due to these conditions (9), being is characterized by abnormal fat accumulation, leading to serious health consequences (10). Various factors contribute to this epidemic, including interactions between biological, behavioral, genetic, and environmental elements (11).

Moreover, obesity increases the risk of developing sleep disorders, cardiovascular risk factors, diabetes, hypertension, high cholesterol, and psychological issues (12). Therefore, combating sedentary behavior has become a global public health problem (13, 14).

There is also growing concern among researchers to alert young people to the dangers of these behaviors, as sedentary habits tend to persist into adulthood (15). Generally, a sedentary lifestyle can be defined as “any waking behavior characterized by an energy expenditure of less than 1.5 metabolic equivalents (METs) while sitting, reclining, or lying" (16). The World Health Organization (WHO) recommends that adolescents spend no more than two hours per day in sedentary behaviors (1). Additionally, the WHO reports that more than 340 million adolescents are obese, and this number is increasing globally (17). Adolescents represent one of the main target groups when considering inactive behaviors (18). Evidence suggests that a sedentary lifestyle is associated with the development of diseases, obesity, and psychological disorders (19) and that inactive habits and obesity are correlated with depression in adolescents (18, 20). Current studies also demonstrate that other emotional issues may arise as a result of obesity Mood states have been studied in various contexts, such as exergames, sports, and adolescents’ lifestyle profiles, including sedentary and active individuals as well as young athletes, showing mood state to be a relevant variable alongside other factors such as sleep and mental health (6–9). Along with high rates of sedentary behaviors, a decline in mood state has also been reported, with an increase in the four negative subscales of the Profile of Mood States (POMS): anger, fatigue, depression, and confusion (6, 10).

Studies suggest that higher levels of PA are associated with better mood states in children and adolescents (11–14). Data indicate that total scores for depression, confusion, anger, and fatigue in the moderate and high PA groups are significantly lower than in the low PA group. Moreover, vigor and self-esteem are increased in individuals who engage in PA (14). Thus, PA may be a useful approach for improving mood in children and adolescents. However, the low quality and limited number of studies restrict the accuracy of these findings (13). Given the relevance of the topic and the current need to gather more information on these relationships, the aim of the present study was to analyze the association between PA, BMI, and mood state in schoolchildren and adolescents.

## 2 Methods

### 2.1 Survey design and study population

This is a cross-sectional population-based study with two thousand seven hundred and fifty-seven school children and adolescents (one thousand three hundred and two boys, one thousand four hundred and fifty-five girls) (aged 14.83 ± 1.67 years) selected in proportion to the size of southern Brazil. All participants were students from the 9th grade of elementary school to the 3rd year of high school, from public institutions administered by the State of Santa Catarina. Ethical approval was obtained from the State University of Santa Catarina (approval number 502.531) and the study participants signed an informed consent form. The sample size was calculated according to the procedures described by 1,4, considering a confidence interval of 95.0% and an error of 3.0%. The prevalence was estimated at 50.0%. When using the school cluster approach, a design effect of 1.5 was considered, which represents an increase of 50.0% in the sample. The sample was derived from 26 different public schools in eight cities in southern Brazil.

### 2.2 Measurement

#### 2.2.1 Sociodemographic and school characterization

A questionnaire was used to characterize the participants, consisting of open and closed questions, with information on sex, age, weight, height, and study period.

#### 2.2.2 Physical activity (PA)

For the practice of PA, the PA questionnaire for children and adolescents was used (16), through the question “Have you practiced sport or physical exercise in clubs, gyms, sports schools, parks, streets, or at home in the last 12 months? (yes or no). Although it is not possible to verify how many times per week, for how long, and not to identify what type of physical activity the participant performed, information based on self-reports on the practices of physical activities performed has been widely used in the literature, which makes it possible to identify whether the participant investigated is active or inactive (21, 22).

#### 2.2.3 Nutritional status

Body mass index (BMI) was used to assess nutritional status. Height and weight were self-reported by the participants and used to calculate the BMI (kg/m^2^).

Children and adolescents were divided and classified based on their BMI, according to the recommendation of the World Health Organization. 17 For the analysis, the classification was divided into 4 groups: BMI ≥ 18.5 Underweight; 18.6–24.5 Normal weight; 25.0–29.9 Overweight; 30.0–40.0 Obesity.

#### 2.2.4 Brunel mood scale

The instrument used to assess mood was the Brunel Mood Scale. This instrument is a Likert scale composed of 24 items, with five response options, ranging from nothing (0), slightly (1), moderately (2), quite (3), to extremely (4). The participant chooses the option that best fits their mood at that moment. The Brunel Mood Scale assesses six dimensions of mood, classified into psychological (depression, anger, and mental confusion) and psychosomatic dimensions (fatigue, tension, and vigor). The score of each dimension ranges from 0 to 16 points; the higher the score, the larger the dimension. A pilot study was conducted to identify the Cronbach's α (alpha) value. Considering our population, Brazilian adolescents in a school environment, the result revealed that the BRUMS is a reliable instrument to measure the six domains of mood state in adolescents (*α* = 0.781).

### 2.3 Procedures

Authorization to carry out the research was obtained from the Secretary of Education of Santa Catarina. Data collection procedures were performed by two trained researchers during physical education classes. Instructions were provided prior to the application of the questionnaire. The variables were collected through standardized printed questionnaires. The participants were informed about the guarantee of anonymity and confidentiality.

### 2.4 Statistical analysis

Data analysis was performed in SPSS using Windows version 20.0. The data were analyzed in two stages. Initially, descriptive analyses were performed (mean ± standard deviation, absolute and relative frequency) and later inferential analyses were performed. The Kruskal–Wallis test was used to compare more than two groups and the Spearman test to verify possible correlations between the variables. Pearson's chi-square test was used to verify the association between PA and mood variables. Poisson regression and simple linear regression analysis were also used to verify the association between PA, BMI, and mood. The level of significance adopted was *p* < 0.05.

## 3 Results

The study included 2,757 students, boys (*n* = 1,455, 52.8%) and girls (*n* = 1,302, 47.2%), between 10 and 19 years of age (mean: 14.83 ± 1.67 years), who attended elementary school (*n* = 1,233, 44.7%) and high school (*n* = 1,524, 55.3%) of the State Education Network of Greater Florianópolis/SC. Table 1 presents the characterization of the sample (Table 1).

**Table 1:** Characterization and categorization variables by BMI of the sample.

Variable	Underweight *N* (575) mean ± SD	Normal weight *N* (1,726) mean ± SD	Overweight *N* (231) mean ± SD	Obesity *N* (61) mean ± SD
Sex (f)				
Male	255 (44.3%)	828 (48%)	117 (50.6%)	31 (50.8%)
Female	320 (56.7%)	898 (52%)	114 (49.4%)	30 (49.2%)
Age	14.11 ± 1.6	15.6 ± 1.5	15.26 ± 1.6	15.7 ± 1.5
Age group (f)				
10–12 years	78 (41.9%)	78 (41.9%)	7 (3.8%)	1 (0.5%)
13–16 years	433 (21.6%)	1,265 (63.2%)	154 (7.7%)	34 (1.7%)
17–19 years	64 (11.2%)	383 (67.2%)	70 (12.6%)	26 (4.6%)
Weight (kg)	45.15 ± 6.3	58.33 ± 9.0	73.65 ± 9.1	96.72 ± 28.0
Height (cm)	162.6 ± 9.7	165.0 ± 10.1	165.5 ± 9.8	165.4 ± 12.6
BMI	17.01 ± 1.2	21.30 ± 1.8	26.81 ± 1.5	35.19 ± 9.2
AF (f)				
Yes	404 (70.3%)	1,326 (76.8%)	168 (72.7%)	45 (73.8%)
No	171 (29.7%)	400 (23.2%)	63 (27.3%)	16 (26.2%)

N, number of participants in each group; SD, standard deviation; (f), frequency of cases; PA, physical activity.

The correlation results between the variables of BMI and mood showed that the increase in BMI is associated with depression, anger, and fatigue in this population. In addition, the results indicate that PA is associated with improved vigor in schoolchildren (*p* < 0.001) (Table 2).

**Table 2:** Associations between BMI, PA and mood variables.

Mood state variables	BMI	Physical activity
Tension	*p* = 0.462 *r* = 0.015	*p* = 0.990 *χ*^2^ = 16.292
Depression	*p* = 0.002^a^ *r* = 0.064	*p* = 0.509 *χ*^2^ = 31.161
Anger	*p* = 0.021^a^ *r* = 0.047	*p* = 0.999 *χ*^2^ = 12.298
Vigor	*p* = 0.255 *r* = −0.023	*p* = 0.000^b^ *χ*^2^ = 78.510
Fatigue	*p* = 0.016^a^ *r* = 0.050	*p* = 0.875 *χ*^2^ = 23.116
Confusion	*p* = 0.312 *r* = 0.021	*p* = 0.765 *χ*^2^ = 25.967

^a^ Spearman correlation significant.

^b^ Significant Pearson's chi-square test.

In the comparative analysis (Table 3), there was a difference between the BMI groups (underweight, normal weight, overweight, and obesity) in the mood states depression and vigor. The overweight (4.06 ± 4.3) and obesity (3.85 ± 4.0) groups presented the highest rates of depression (*p* = 0.004). The obesity group showed greater vigor (*p* = 0.047).

**Table 3:** Differences in mood between BMI rating groups.

Mood state variables	Underweight	Normal weight	Overweight	Obesity	*P* value
Tension	4.77 ± 3.8	4.51 ± 3.7	4.83 ± 3.7	5.29 ± 3.5	0.285
Depression	2.83 ± 3.5^b^	2.95 ± 3.6^b^	4.06 ± 4.3^b^	3.85 ± 4.0	0.004^a^
Anger	3.70 ± 4.2	3.60 ± 4.0	4.41 ± 4.4	3.77 ± 3.7	0.085
Vigor	8.96 ± 4.2	8.64 ± 4.3^b^	8.00 ± 4.3^b^	9.27 ± 3.7	0.047^a^
Fatigue	4.95 ± 4.0	5.13 ± 4.1	5.81 ± 4.3	5.41 ± 4.3	0.186
Confusion	4.06 ± 3.7	3.84 ± 3.5	4.31 ± 3.6	4.60 ± 3.8	0.097

^a^ Statistically significant difference. Teast of Kruskal–Wallis.

^b^ *post hoc* Dunn: Vigor (normal weight * overweight—*p* = 0.006); depression (underweight * overweight; normal weight *Overweight—*p* = 0.001).

Regarding mood, differences were observed between male and female sexes, and between active and inactive individuals, in depression, anger, vigor, fatigue, and confusion (Table 4).

**Table 4:** Differences in mood between genders and PA practice.

Variable	Tension	Depression	Anger	Vigor	Fatigue	Confusion
Sex	0.283	0.000^a^	0.002^a^	0.000^a^	0.000^a^	0.000^a^
Female	4.53 ± 3.7	3.10 ± 3.6	3.72 ± 4.0	7.87 ± 4.3	5.47 ± 4.2	4.14 ± 3.6
Male	4.30 ± 3.5	2.68 ± 3.5	3.40 ± 4.0	9.43 ± 4.2	4.70 ± 3.9	3.60 ± 3.4
Physical activity	0.812	0.001^a^	0.029^a^	0.000^a^	0.016^a^	0.000^a^
Active	4.41 ± 3.6	2.86 ± 3,5	3.56 ± 4.1	8.91 ± 4.3	5.08 ± 4.0	3.82 ± 3.5
Inactive	4.47 ± 3.7	3.04 ± 3.6	3.62 ± 4.0	7.66 ± 4.3	5.23 ± 4.2	4.11 ± 3.6

In school children and adolescents.

^a^ Statistically significant difference. Wilcoxon test.

The results of the linear regression indicate that inactive individuals were 41.1% more likely to have the mood state depression (F = 6.371; *p* = 0.012; *R*^2^ = 0.003; B = −0.411), 41.5% more likely to be angry (F = 5.094; *p* = 0.024; *R*^2^ = 0.002; B = −0.415), 42.4% more chances of fatigue (F = 5.370; *p* = 0.021; *R*^2^ = 0.002; B = −0.424), and 52.7% more chances of mental confusion than active individuals (F = 10.969; *p* = 0.001; *R*^2^ = 0.004; B = 0.527). PA may explain 39% of vigor in this population (F = 103.062; *p* = 0.000; *R*^2^ = 0.039; B = −1.909), and active children and adolescents presented 2 times more vigor than inactive ones.

**Figure 1:**
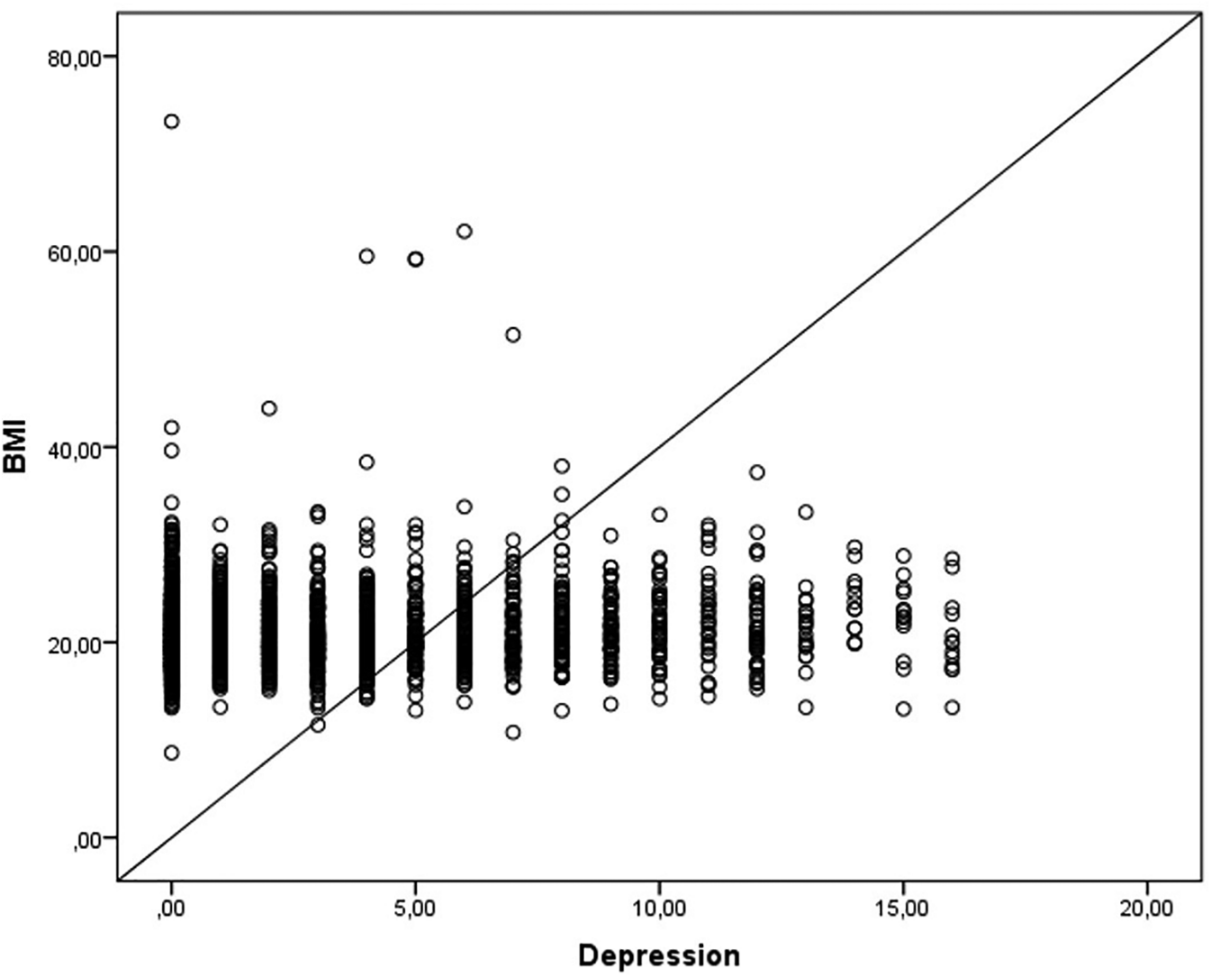
Presents the relationship between BMI and depression in this population.

Regarding BMI, it was observed that individuals with normal weight have a 1.4 times lower chance of presenting depression when compared to obese individuals (F = 10.958, *p* = 0.000; *R*^2^ = 0.009; B = 1.401) (Figure 1).

Males when compared to females are 35.4% less likely to have depression (F = 5.788, *p* = 0.016; *R*^2^ = 0.002; B = −0.354), 41.3% less chances of anger (F = 6.143, *p* = 0.013; *R*^2^ = 0.002; B = −0.413), 66% less chances of fatigue (F = 15.887, *p* = 0.000; *R*^2^ = 0.006; B-0.660), 55.5% less chances of mental confusion (F = 14.869, *p* = 0.000; *R*^2^ = 0.005; B = −0.555), and 1.6 times more vigor than females (F = 94.059, *p* = 0.000; *R*^2^ = 0.035; B = 1.648).

Regarding Poisson regression, in the crude analysis there was an association between PA and mood variables. In the adjusted analysis, the same variables remained associated with the outcome, revealing that the probability of being physically active increases for each increase of one unit of vigor (PR = 1.09; CI95% = 1.07–1.11) and decreases for each increase in a confounding unit (PR = 0.95; CI95% = 0.92–0.98).

## 4 Discussion

The current study aimed to analyze the association between PA, BMI, and mood state in school children and adolescents. In this sense, it was found that the overweight and obesity groups presented the highest rates of the mood state depression. In addition, individuals with a higher BMI are more likely to present changes in mood, with higher rates of depression, anger, and fatigue. It is already established in the literature that changes in mood states impact mental health (19). Evidence the association between obesity and depression in children and adolescents and the data are alarming, requiring special attention in the development of actions and public policies.

Our study considered the characteristics of the subgroups of the sample investigated, observing differences in mood states between sexes, and between active and inactive individuals. Changes in depression, anger, vigor, fatigue, and confusion were observed, and male children and adolescents were less likely to develop mood changes compared to females. Regarding emotions, other findings suggest that there are sex differences and increased risk of depression and anxiety symptoms (20). Results of previous studies defend a context-dependent role of sex hormones in the formation of susceptibility to depression (23) highlighting social stress as a potent risk factor for depression, particularly for women with greater sensitivity to hormonal fluctuation. According to Slavich and Sacher, women seem to have a relatively higher risk of depressed mood than men, and this is especially true for those who are in the middle of a period of hormonal transition, as is the case in the population studied. These data reinforce the need for longitudinal studies that examine sex differences in disorders related to mood disorders (24).

Inactive individuals, on the other hand, have higher rates of depression, anger, fatigue, and confusion than active individuals. Thus, the practice of PA seems to promote benefits, being associated with improved vigor in this population. Children and adolescents who practiced PA presented less depression, anger, fatigue, and confusion and twice as high vigor as those who were inactive. In a survey conducted with a thousand children and adolescents during the pandemic, it was found that more PA and less screen time are associated with better mental health for children (25).

In the systematic review of Andrade et al. (6), similar results were verified in obese adolescents practicing active electronic games, as in PA practice, positive effects were observed related to psychological aspects (7). The study by Matias et al. concluded that there are associations between exposure to PA practices and better mood profiles in adolescence, similar to the results found in this study (26). However, studies have shown that obese adolescents present higher levels of depression (27) body dissatisfaction when compared to children and adolescents with normal weight (10, 28). This result is in agreement with the present study, where it was observed that individuals with normal weight have 1.4 less chances of presenting mood depression in relation to obese individuals.

Vidmar et al. presented similar results when investigating the differences between obese adolescents with and without food dependence in the symptoms of depression and stress (29). Obese adolescents diagnosed with food dependence presented more depressive symptoms and greater perceived stress. According to the authors, emotional difficulties may increase the challenges of adhering to weight control recommendations and may lead to greater distress and justify multidisciplinary support (29).

In the present study, the overweight and obesity groups presented the highest rates of depression, but surprisingly the obesity group presented greater vigor. A possible explanation for this result is the fact that this group, because it presents the need for weight reduction, included a high percentage of PA practitioners (73.8%). Regarding the psychological consequences of overweight and obesity, evidence points to a negative perception of the body, as well as low self-esteem (10, 30). These factors may subsequently reduce the motivation to participate in leisure-time physical activities, contributing to the establishment of inactivity and reducing the perception of physical competence (10, 30). concluded that the perception of physical competence differs according to the nutritional status of adolescents, and lower perceptions of aerobic resistance and flexibility favor the development of overweight and obese adolescents (15).

According to Cardel et al. increasing PA has the potential to improve cardiometabolic outcomes and is predictive of sustained weight change 10 years after involvement in a weight loss intervention in adolescents (31). In our study, it was observed that the probability of being physically active increases for each increase in a unit of vigor, and PA can explain 39% of the vigor in this population. Thus, it is suggested that children and adolescents perform 30 min of moderate-intensity PA per day, 5 days a week, or 20 min of vigorous PA on 3 days a week. Furthermore, physical inactivity should be reduced by limiting non-academic screen time and other sedentary activities to less than 2 h per day. It is known that PA interventions at school and in the community as part of an obesity prevention or treatment program can benefit the executive functions of obese or overweight children (32, 33).

### 4.1 Limitations and future studies

Although our population-based cross-sectional study was developed with 2,757 school children and adolescents, an important sample, the study has limitations regarding the evaluation of the PA measure, that was self-reported, which may imply some inaccuracy, although quality international studies use this method.

Future studies should be undertaken directly and objectively to evaluate the practice of exercise, to obtain data on possible motivations and associate the findings with overweight and obesity in children and adolescents, as well as to analyze other psychological variables, such as depression, anxiety, and stress, directly involved in exercise practices and in weight and obesity in youth.

### 4.2 Strength, innovations and applications

Our study investigated a representative sample of children and adolescents from southern Brazil, to analyze the association between PA, body mass index (BMI), and mood state. Studies associating exercise level, and overweight and obesity of adolescents in relation to mood are scarce, which is an innovation of the study. The results of psychological (depression, anger, and mental confusion) and psychosomatic (fatigue, tension, and vigor) variables due to exercise and overweight and obesity contribute to better understanding of the problem and assist in interventions with exercise, to apply better strategies and content that take into account mood factors as important for participation, adherence, and improved results of treatments and exercise programs, especially in this post-Covid19 pandemic scenario where body exercise and sports practices have been significantly impaired. Physical education professionals, physical therapists, sports physicians, and sports and health sciences in general can benefit from the knowledge and analysis for research and intervention.

## 5 Conclusion

The results of the present study indicate a significant association between mood disturbances and overweight and obesity in children and adolescents. Individuals with higher BMI values showed notable changes in mood, particularly increased levels of depression, anger, and fatigue. Additionally, males were found to be less prone to mood fluctuations compared to females. Inactive individuals exhibited higher levels of depression, anger, fatigue, and confusion when compared to their active counterparts.

Furthermore, the practice of physical activity (PA) was shown to have a positive effect on mood, with active children and adolescents reporting lower levels of depression, anger, fatigue, and confusion, and higher levels of vigor. These findings underscore the importance of PA as a protective factor against mood disturbances in this population, highlighting its potential role in improving emotional well-being.

## Data Availability

The raw data supporting the conclusions of this article will be made available by the authors, without undue reservation.
